# Association of Mental Health Conditions, Recent Stressful Life Events, and Adverse Childhood Experiences with Postpartum Substance Use ― Seven States, 2019–2020

**DOI:** 10.15585/mmwr.mm7216a1

**Published:** 2023-04-21

**Authors:** Andrea Stewart, Jean Ko, Beatriz Salvesen von Essen, Madison Levecke, Denise V. D’Angelo, Lisa Romero, Shanna Cox, Lee Warner, Wanda Barfield

**Affiliations:** ^1^Division of Reproductive Health, National Center for Chronic Disease Prevention and Health Promotion, CDC; ^2^Epidemic Intelligence Service, CDC; ^3^Oak Ridge Institute for Science and Education, Oak Ridge, Tennessee; ^4^Division of Violence Prevention, National Center for Injury Prevention and Control, CDC.

Most pregnancy-related deaths due to mental health conditions, which include overdose and poisoning related to substance use disorder, occur during the late (43–365-day) postpartum period (*1*). Adverse childhood experiences and stressful life events are associated with increased substance use during pregnancy (*2*,*3*). Pregnancy Risk Assessment Monitoring System (PRAMS) respondents in seven states with high opioid overdose mortality rates were recontacted 9–10 months after giving birth in 2019 and asked about postpartum prescription opioid misuse,* tobacco use, unhealthy alcohol use,^†^ and use of other substances.^§^ Substance and polysubstance use prevalence estimates were calculated, stratified by mental health and social adversity indicators. Overall, 25.6% of respondents reported postpartum substance use, and 5.9% reported polysubstance use. The following conditions were associated with higher substance and polysubstance use prevalence in postpartum women: depressive symptoms, depression, anxiety, adverse childhood experiences, and stressful life events. Substance use prevalence was higher among women who experienced six or more stressful life events during the year preceding the birth (67.1%) or four adverse childhood experiences related to household dysfunction (57.9%). One in five respondents who experienced six or more stressful life events in the year before giving birth and 26.3% of women with four adverse childhood experiences reported postpartum polysubstance use. Clinical and community- and systems-level interventions to improve postpartum health can include screening and treatment for depression, anxiety, and substance use disorders during the postpartum period. Evidence-based strategies can prevent adverse childhood experiences and mitigate the immediate and long-term harms.^¶^

PRAMS is a collaboration between CDC and participating jurisdictions to conduct population-based surveillance for maternal experiences before, during, and after pregnancy among women with a recent live birth (*4*). To better understand opioid use and risks for opioid use among postpartum women, PRAMS recontacted respondents from 2019 in seven states with high rates of opioid-involved overdose deaths** 9–10 months after they gave birth. They were asked about use of opioid pain relievers since giving birth, including source and reasons for use, as well as use of tobacco, alcohol, and other substances^††^; current depression or anxiety^§§^; and depressive symptoms during the previous 30 days.^¶¶^ Respondents were also asked whether they had experienced any of four household-dysfunction adverse childhood experiences before age 18 years.*** During the core PRAMS survey, respondents in six of the seven states were asked whether they had experienced any of 14 stressful life events during the year preceding the birth.^†††^

Postpartum substance use was defined as the misuse of opioid pain relievers, unhealthy alcohol use, or any use of tobacco or other substances since giving birth. Polysubstance use was defined as using two or more of these substances. Data were weighted to account for the PRAMS sample, nonresponse, and noncoverage. Estimates are representative of women with a live birth in each participating state during a 5-month period in 2019.^§§§^

Weighted prevalence and 95% CIs of postpartum substance and polysubstance use were estimated overall and by age, combined race and ethnicity, education level, health insurance status at the time of recontact, state of residence, previous 30-day depressive symptoms, current depression, current anxiety, number of adverse childhood experiences, and number of stressful life events. Chi-square tests with adjusted Wald F statistics were used to determine statistical significance of differences across characteristics. Multivariable logistic regression models adjusted for age, race and ethnicity, education level, health insurance status, and state of residence were used to calculate adjusted prevalence ratios (aPRs) of postpartum substance and polysubstance use for each mental health and social adversity indicator. Analyses were conducted using SAS-callable SUDAAN (version 11.0; RTI International). This study was reviewed and approved by the Institutional Review Boards at CDC and each participating PRAMS site.^¶¶¶^

Among 1,990 respondents in seven states, 1,920 (96%) provided data on postpartum substance use. The weighted prevalences of postpartum substance use and polysubstance use were 25.6% and 5.9%, respectively. Substance use prevalence varied significantly across categories of education level, health insurance status, and state of residence. Significant differences in polysubstance use rates were observed by race and ethnicity and health insurance status (Table 1).

**TABLE 1:** Prevalence of postpartum substance use, by maternal characteristics, depressive symptoms, self-reported depression and anxiety, and number of adverse childhood experiences — Pregnancy Risk Assessment Monitoring System, seven states, 2019

Characteristic	No., unweighted	Any postpartum substance use* % (95% CI)	Postpartum polysubstance use* % (95% CI)
**Total**	**1,920**	**25.6 (22.8–28.7)**	**5.9 (4.6–7.6)**
**Age group, yrs**			
≤19	63	31.2 (16.5–51.0)	3.6 (1–11.6)
20–24	299	34.3 (26.9–42.5)	10.6 (6.3–17.4)
25–34	1,162	24.4 (20.9–28.4)	5.2 (3.9–7.0)
≥35	396	21.4 (15.9–28.3)	4.9 (2.2–10.3)
**Race and ethnicity^†^**			
Black, non-Hispanic	352	23.0 (16.8–30.6)	4.6 (2.4–8.8)
Hispanic or Latino	244	20.4 (13.3–29.9)	3.1 (1.4–6.7)
White, non-Hispanic	1,157	27.6 (24.0–31.6)	7.0 (5.2–9.3)
Other, non-Hispanic	143	16.4 (10.0–25.7)	2.1 (0.9–4.6)
**Education level, yrs^§^**			
<12	179	30.1 (20.5–41.8)	3.7 (1.2–10.6)
12	429	35.4 (29.0–42.5)	10.0 (6.5–15.1)
>12	1,281	21.8 (18.5–25.5)	4.9 (3.5–6.8)
**Health insurance status 9–10 mos after giving birth^†,§^**			
Private^¶^	1,085	21.1 (17.6–25)	4.4 (2.8–6.7)
Medicaid	616	37.6 (31.8–43.7)	8.6 (6.1–12.1)
None	173	16.7 (10.5–25.6)	9.0 (4.7–16.5)
Other	40	21.2 (9.7–40.5)	2.8 (0.5–13.5)**
**State of residence^§^**			
Kentucky	316	30.7 (23.9–38.5)	8.9 (5.3–14.3)
Louisiana	273	25.2 (18.6–33.2)	3.9 (1.8–8.1)
Massachusetts	364	24.8 (19.5–30.9)	6.5 (3.8–10.8)
Missouri	272	31.8 (25.0–39.5)	7.1 (4.0–12.4)
Pennsylvania	219	22.9 (16.7–30.7)	4.5 (2.2–9.1)
Utah	297	17.8 (13.0–24.0)	6.4 (3.5–11.5)
West Virginia	179	31.2 (22.9–40.8)	5.1 (3.9–6.7)
**Depressive symptoms during previous 30 days^†,§,††^**			
Yes	152	48.5 (36.7–60.6)	17.5 (9.4–30.3)
No	1,753	24.0 (21.2–27.1)	7.0 (3.4–13.7)
**Current depression^†,§^**			
Yes	347	42.8 (35.0–51.1)	17.3 (11.8–24.6)
No	1,572	21.9 (19.0–25.1)	3.5 (2.5–4.7)
**Current anxiety^†,§^**			
Yes	580	40.9 (35.0–47.1)	13.0 (9.6–17.2)
No	1,338	18.9 (15.9–22.3)	2.8 (1.8–4.6)
**No. of adverse childhood experiences^†,§,§§^**			
0	835	16.6 (13.1–20.8)	2.0 (1.1–3.7)
1	564	24.8 (19.7–30.8)	5.0 (2.8–8.9)
2–3	401	39.5 (32.6–46.9)	11.9 (8.2–17.1)
4	76	57.9 (42.1–72.3)	26.3 (14.5–43)
**No. of stressful life events in yr before giving birth^†,§,¶¶^**			
0	496	17.0 (12.5–22.7)	1.9 (0.9–4.0)
1–2	722	20.3 (16.1–25.1)	5.0 (3.0–8.4)
3–5	401	37.4 (30.9–44.4)	9.6 (6.6–13.8)
≥6	109	67.1 (54.2–77.8)	20.8 (12.2–33.3)

**Abbreviation:** PRAMS = Pregnancy Risk Assessment Monitoring System.

* Defined as self-reported excessive alcohol use (eight or more drinks per week) or any binge drinking (more than four drinks in a single 2-hour episode); opioid misuse (taking prescription opioid medication for reason other than pain or obtained from a source other than a health care provider); or any use of tobacco, marijuana products (including cannabidiol and synthetic marijuana such as K2 or Spice), heroin, cocaine, amphetamines, hallucinogens, tranquilizers, or inhalants since giving birth. Polysubstance use indicates use of two or more of these substances.

^†^ p<0.05 for an adjusted Wald F chi-square test of differences across categories for “polysubstance use.”

^§^ p<0.05 for an adjusted Wald F chi-square test of differences across categories for “any substance use.”

^¶^ Includes Tricare or other military health care.

** Indicates unweighted denominator count <60; estimate might be unstable.

^††^ Answering “always” or “often” to either of the Patient Health Questionnaire-2 questions: “How often have you felt down, depressed, or hopeless?” and “How often have you had little interest or little pleasure in doing things you usually enjoyed?”

^§§^ Parental separation/divorce, living with someone who was a problem drinker/alcoholic or using drugs, living with someone with mental illness, or living with someone who was incarcerated.

^¶¶^ Collected during the core PRAMS survey 2–6 months after a live birth. West Virginia data were not available. Unweighted n = 1,728. The full list of events is available in the PRAMS Standard Question list. https://www.cdc.gov/prams/questionnaire.htm

The prevalence of substance use was approximately twice as high among respondents who reported the following conditions than among those who didn’t: depressive symptoms (48.5% versus 24.0%), current depression (42.8% versus 21.9%), and anxiety (40.9% versus 18.9%) (Table 1). The prevalence of polysubstance use was also higher among respondents who reported those conditions than among those who did not: 17.5% versus 7.0%, 17.3% versus 3.5%, and 13.0% versus 2.8%, respectively.

Among respondents who reported all four household-dysfunction adverse childhood experiences, the prevalence of postpartum substance use was 57.9% and of polysubstance use was 26.3%. Among respondents reporting six or more stressful life events during the year before giving birth, the prevalence of postpartum substance use was 67.1% and of polysubstance use was 20.8%.

Adjusted prevalences of postpartum substance use and polysubstance use among respondents with depressive symptoms were 1.8 and 3.0 times as high, respectively, as among those without these symptoms. aPRs for the association of any postpartum substance and polysubstance use with current depression and anxiety were similar (Table 2). Prevalences of postpartum substance and polysubstance use among those reporting two to four adverse childhood experiences were 2.1 and 5.5 times as high, respectively, as among those reporting none. Compared with the prevalence of substance use among those who reported no stressful life events in the year before giving birth, prevalence of substance use was 3.6 times as high and of polysubstance use was 9.1 times as high among those who reported six or more stressful life events (Table 2).

**TABLE 2:** Association of mental health conditions, recent stressful events, and adverse childhood experiences with postpartum substance use — Pregnancy Risk Assessment Monitoring System, seven states,* 2019

Characteristic	Any postpartum substance use, aPR^†^ (95% CI)	Postpartum polysubstance use, aPR^†^ (95% CI)
**Depressive symptoms during previous 30 days**		
No	Ref	Ref
Yes	1.8 (1.3–2.5)	3.0 (1.4–6.2)
**Current depression**		
No	Ref	Ref
Yes	1.7 (1.3–2.2)	4.5 (2.7–7.5)
**Current anxiety**		
No	Ref	Ref
Yes	1.9 (1.5–2.4)	3.9 (2.1–7.1)
**No. of adverse childhood experiences**		
0	Ref	Ref
1	1.3 (0.9–1.7)	2.1 (0.9–5.3)
2–4^§^	2.1 (1.5–2.7)	5.5 (2.6–11.4)
**No. of stressful life events^¶^ in yr before giving birth**		
0	Ref	Ref
1–2	1.2 (0.8–1.7)	2.8 (1.1–7.2)
3–5	1.9 (1.4–2.7)	4.3 (1.8–10.6)
≥6	3.6 (2.5–5.1)	9.1 (3.4–24.7)

**Abbreviations:** aPR = adjusted prevalence ratio; Ref = referent group.

* Kentucky, Louisiana, Massachusetts, Missouri, Pennsylvania, Utah, and West Virginia.

^†^ Models were adjusted for age, race and ethnicity, education level, health insurance status, and state of residence. aPRs were estimated using separate models for each mental health and psychosocial indicator. ^§^ Categories were combined because of small numbers of observations preventing estimation of aPRs.

^¶^ Data on stressful life events were not available for West Virginia.

## Discussion

In a population-based sample of women from seven states with high rates of opioid overdose deaths who had participated in a PRAMS survey and were recontacted 9–10 months after a live birth in 2019, approximately one in four reported substance use, and more than one in 17 reported polysubstance use since giving birth. These results update postpartum polysubstance use estimates for the United States and demonstrate that polysubstance use commonly co-occurs during the postpartum period with mental health conditions and a history of predelivery life stressors and adverse childhood experiences. In the 2006–2014 National Survey of Drug Use and Health (NSDUH), 5.1% of pregnant women and 24.3% of nonpregnant women reported using more than one substance during the previous month (*5*). In the 2009–2019 NSDUH, previous-month polysubstance use during pregnancy ranged from 31.3% in the first trimester to 7.8% in the third trimester (*6*). NSDUH and PRAMS use different sampling and administration methods as well as different definitions of alcohol use; therefore, direct comparisons between the two surveillance systems is difficult.

A history of stressful life events in the year before giving birth and adverse childhood experiences related to household dysfunction were both associated with a higher prevalence of postpartum substance use. This finding is consistent with studies of perinatal alcohol (*2*) and marijuana use (*3*). The American College of Obstetrics and Gynecologists (ACOG) recommends a comprehensive postpartum visit within 12 weeks of giving birth that includes 1) screening for depression, anxiety, and substance use disorder; 2) assessment of material needs such as housing, utilities, and food; and 3) referrals for follow-up care and resources (*7*,*8*). ACOG also emphasizes that postpartum care should be a process tailored to individual needs, coordinated between obstetric and primary care providers (*7*). The United States Preventive Services Task Force provides screening guidance for unhealthy substance use and depression, including for pregnant and postpartum women (*9*,*10*). Approaches to reduce postpartum harms of adverse childhood experiences include family-centered treatment approaches for substance-use disorders and victim-centered services.

Recommendations from Maternal Mortality Review Committee (MMRC) investigations of pregnancy-related mental health and substance use–associated deaths highlight potential community- and systems-level interventions (*1*). To prevent maternal deaths, MMRC recommends improving social, family, and peer support and education for patients, providers, and communities (*1*). Other strategies to prevent, identify, and improve access to treatment of opioid use disorder among pregnant and postpartum women include sharing best practices through collaborative learning communities, and perinatal quality collaboratives supporting multidisciplinary teams to build capacity for screening, treating, and coordinating care for pregnant and postpartum women with opioid use disorder and their infants.****

The findings in this report are subject to at least five limitations. First, these data are only generalizable to the seven states that administered the callback survey, where overdose mortality rates were high. Second, substance use was self-reported and is subject to social desirability bias and therefore might be underestimated. Third, misuse of prescription medications other than opioid pain relievers was not included in the definition of substance misuse. Fourth, information on adverse childhood experiences such as physical, emotional, or sexual abuse, or stressful life events after giving birth, was not collected. Finally, the definition of polysubstance use in this report represents use of two or more substances any time since giving birth and not necessarily that respondents used multiple substances at the same time.

Substance use was common in the postpartum period, particularly among those women who reported experiencing social adversity. Comprehensive prenatal and postpartum care, including screening and treatment for mental health and substance use disorders and community support and education, might help address postpartum substance use–related morbidity and mortality.

**Summary**

**What is already known about this topic?**

Most pregnancy-related deaths due to mental health conditions, including substance use disorder–related overdose and poisoning, occur during the late (43–365-day) postpartum period.

**What is added by this report?**

In seven states with high opioid-involved overdose mortality rates, depressive symptoms, depression, anxiety, adverse childhood experiences, and stressful life events were associated with higher substance and polysubstance use prevalences among postpartum women. Postpartum substance use prevalence was most common among women experiencing six or more stressful life events in the year before giving birth (67.1%) or four household-dysfunction adverse childhood experiences (57.9%).

**What are the implications for public health?**

Clinical and community- and systems-level interventions can address postpartum substance use and mental health conditions and lessen harms associated with adverse childhood experiences.

## Footnotes

* Opioid misuse was defined as taking prescription opioid medication for a reason other than pain or obtained from a source other than a health care provider. https://www.cdc.gov/mmwr/volumes/69/wr/mm6928a1.htm

† Meeting the CDC definition of binge drinking (more than four drinks during a single 2-hour episode) or excessive alcohol use (eight or more drinks per week) since giving birth.

§ Including heroin, marijuana products, cocaine, amphetamines, hallucinogens, tranquilizers, or inhalants.

¶
https://www.cdc.gov/violenceprevention/pdf/preventingACES.pdf

** Kentucky, Louisiana, Massachusetts, Missouri, Pennsylvania, Utah, and West Virginia.

†† Respondents were asked “Since your baby was born, have you taken or used __________?” with the following options: “Cannabidiol or CBD products”; “Marijuana or hash”; “Synthetic marijuana, or K2 or Spice”; “Heroin, also known as smack, junk, Black Tar, or Chiva”; “Amphetamines, also known as uppers, speed, crystal meth, crank, ice, or agua”; “Cocaine, also known as crack, rock, coke, blow, snow, or nieve”; “Tranquilizers, also known as downers or ludes”; “Hallucinogens, such as LSD/acid, PCP/angel dust, Ecstasy, Molly, mushrooms, or bath salts”; and “Sniffing gasoline, glue, aerosol spray cans, or paint to get high, also known as huffing.”

§§ Part of a series of questions that started, “I’m going to read a list of health conditions. For each one, please tell me if you currently have it. Do you have ____?”

¶¶ Answering “always” or “often” to either of the Patient Health Questionnaire-2 questions: “How often have you felt down, depressed, or hopeless?” and “How often have you had little interest or little pleasure in doing things you usually enjoyed?”

*** Parental separation/divorce, living with someone who was a problem drinker/alcoholic or using drugs, living with someone with mental illness, or living with someone who was incarcerated.

††† Sick and hospitalized family member, separation/divorce, moved, experiencing lack of housing, lost job, partner lost job, partner or self had hours or pay cut, extended time apart from partner due to military deployment or work-related travel, argued with partner more than usual, partner said they didn't want them to be pregnant, difficulty paying bills, partner or self went to jail, someone very close had problem with substances, or someone very close died. Data was collected during the core PRAMS survey 2–6 months after a live birth. West Virginia data were not available.

§§§ Five months of data were weighted to represent women having a live birth during approximately 5 months in 2019.

¶¶¶ 45 C.F.R. part 46; 21 C.F.R. part 56.

****
https://www.cdc.gov/reproductivehealth/maternalinfanthealth/substance-abuse/opioid-use-disorder-pregnancy/working-with-states-partners-organizations.htm
